# Weight loss surgery for non-morbidly obese populations with type 2 diabetes: is this an acceptable option for patients?

**DOI:** 10.1017/S146342361300025X

**Published:** 2013-06-05

**Authors:** Rachael H. Summers, Helen Elsey, Michael Moore, Christopher Byrne, James Byrne, Richard Welbourn, Paul Roderick

**Affiliations:** 1 Health Services Research Fellow, Primary Care and Population Sciences, Faculty of Medicine, University of Southampton, Southampton, UK; 2 Public Health Speciality Registrar, Academic Unit of Public Health, Leeds Institute of Health Sciences, University of Leeds, Leeds, UK; 3 Reader & Academic Lead Primary Care Research Network South West, Primary Care and Population Sciences, Faculty of Medicine, University of Southampton, Southampton, UK; 4 Professor Endocrinology & Metabolism, Nutrition and Metabolism, Faculty of Medicine, University of Southampton, Southampton, UK; 5 Southampton National Institute for Health Research Biomedical Research Centre, University Hospital Southampton, Southampton, UK; 6 Consultant Surgeon, General Surgery, University Hospital Southampton NHS Foundation Trust, Southampton, UK; 7 Consultant Surgeon, Department of Bariatric Surgery, Musgrove Park Hospital, Taunton, UK; 8 Professor of Public Health, Primary Care and Population Sciences, Faculty of Medicine, University of Southampton, Southampton, UK

**Keywords:** bariatric surgery, obesity, qualitative research, type 2 diabetes

## Abstract

**Aim:**

To explore the views of non-morbidly obese people (BMI 30–40 kg/m^2^) with type 2 diabetes regarding: (a) the acceptability of bariatric surgery (BS) as a treatment for type 2 diabetes, and (b) willingness to participate in randomised controlled trials comparing BS versus non-surgical intervention.

**Background:**

Despite weight management being a key therapeutic goal in type 2 diabetes, achieving and sustaining weight loss is problematic. BS is an effective treatment for people with morbid obesity and type 2 diabetes; it is less certain whether non-morbidly obese patients (BMI 30–39.9 kg/m^2^) with type 2 diabetes benefit from this treatment and whether this approach would be cost-effective. Before evaluating this issue by randomised trials, it is important to understand whether BS and such research are acceptable to this population.

**Methods:**

Non-morbidly obese people with type 2 diabetes were purposively sampled from primary care and invited to participate in semi-structured interviews. Interviews explored participants’ thoughts surrounding their diabetes and weight, the acceptability of BS and the willingness to participate in BS research. Data were analysed using Framework Analysis.

## Introduction

Obesity is one of the leading global risks for mortality worldwide (World Health Organisation, 2009). The National Centre for Health and Statistics (Ogden and Carroll, 2010) indicates that in the United States adult obesity rose from 23% in 1988–1994 to 34% in 2007–2008. Similar trends have been reported in other developed countries, with the United Kingdom having the highest obesity rates of all European countries [Organisation for Economic Co-Operation and Development (OECD), 2010; The NHS Information Centre for Health and Social Care, 2011]. Prevalence and associated burden of morbidity such as type 2 diabetes will continue to rise (Adams *et al*., 2006; Holman *et al*., 2011; Wang *et al*., 2011) without effective intervention.

Weight reduction is a key therapeutic goal in the management of obese patients with type 2 diabetes (Williamson *et al*., 2000; Holman *et al*., 2011), as it decreases fat mass, improves glycaemic control and reduces complications (Buchwald *et al*., 2009; American Diabetes Association, 2011). However, many of the weight management interventions for type 2 diabetes have limited effect (Norris *et al*., 2005; Bailey, 2011). Bariatric surgery (BS) is one strategy to achieve greater and more sustained weight loss. Buchwald *et al*.'s (2009) meta-analysis of 621 studies found that diabetes was resolved in 78% of people, which rose to 86% when including those with improved diabetes control. The UK National Bariatric Surgery Registry published similar data (Welbourn *et al*., 2010). Before surgery, 28% of BS patients had type 2 diabetes, although at two years of post-surgical follow-up, glucose tolerance had improved and diabetes had resolved in 86% of these patients (Welbourn *et al*., 2010). Mingrone *et al*.'s (2012) randomised controlled trial (RCT) compared medical therapy versus surgery in 60 morbidly obese patients with type 2 diabetes, and found that remission had occurred in 75% and 95% of patients in the gastric bypass and biliopancreatic diverson groups, respectively, in comparison with no remissions in the medical therapy group. Consequently, a growing body of evidence supports BS as an intervention for improving and/or correcting type 2 diabetes. Although evidence suggests promising results, most research investigates the effects of BS in people who are morbidly or severely obese (BMI ⩾ 40 kg/m^2^). However, many patients with type 2 diabetes have BMI 30–39 kg/m^2^, and given the limited effectiveness of non-surgical strategies, there is a need to assess the role of BS in such patients. One small RCT involving 60 patients has indicated that the benefits associated with BS also apply in patients with BMI 30–40 kg/m^2^ (Dixon *et al*., 2008). At two years, diabetes remission rates were 73% in surgical arm and 27% in the non-surgical, with 62.5% versus 4.3% loss of excess weight, respectively (Dixon *et al*., 2008). However, this study was relatively small and needs replication. The need for further research is particularly important for countries such as the United Kingdom, which allocate health provision on the basis of best available evidence. In the United Kingdom, current guidelines from the National Institute for Health and Clinical Excellence (NIHR, 2006) only recommend BS for people whose BMI is ⩾35–39 kg/m^2^ in the presence of weight-related conditions, including type 2 diabetes. Moreover, with considerable geographic variation in eligibility criteria between primary care trusts (Haslam, 2010), a stronger evidence base is required to inform decisions on bariatric surgery (BS) provision.

Although evidence into the effectiveness of BS as a treatment for non-morbidly obese people with type 2 diabetes is needed, understanding the patients’ perspectives of BS as an appropriate intervention to control their weight and improve their glycaemic control is vital, providing a sense of population interest in BS and factors that may enhance recruitment to future trials. The evidence exploring patient motivations for undergoing BS suggest the desire to reduce co-morbidities, enhance quality of life and regain a sense of control are key motivating factors (Ogden *et al*., 2006; Munoz *et al*., 2007; Karmali *et al*., 2011). However, just as RCTs investigating BS tend to study morbidly obese populations, qualitative studies into BS usually focus on patients with higher BMIs and largely explore the views of individuals who have already undergone surgery, thereby omitting the perspectives of those unwilling to consider BS or the non-morbidly obese.

The aim of this study was to explore (a) the acceptability of BS as a treatment strategy, and (b) willingness to participate in an RCT comparing BS with a non-surgical strategy, within this group, to elucidate factors associated with willingness to consider BS and participate in research.

## Methods

### Sampling and recruitment

Five general practitioner (GP) clinics (four in the South of England, one in the North) were involved in identifying and inviting eligible patients. GP practices were chosen to give a diversity of catchment areas in terms of urban–rural and geographical location, deprivation and ethnicity. Table 1 provides the study inclusion and exclusion criteria. Patients were purposively sampled on the basis of demographic information: age, sex, BMI, ethnicity and duration of type 2 diabetes. Data collection ended when no new responses were revealed by further interviewing and data saturation was judged to have been achieved.

**Table 1:** Study inclusion and exclusion criteria

Inclusion criteria	Exclusion criteria
Aged between 18 and 74 years	Recent acute coronary heart disease
Diagnosis of type 2 diabetes for 2 years or more	Severe respiratory disease
BMI 30–39.9 kg/m^2^	Recent cancer
BMI 27.5–29.9 kg/m^2^ (lower BMI range for South Asian people only)	Neurological disorders
	Severe mental health conditions
	Other health reason the GP felt would prevent inclusion

### Data collection

Data were collected using semi-structured interviews, which followed an interview guide developed by the study team in consultation with two lay advisors. The guide consisted of open-ended questions relating to one of four topic areas: diabetes and diabetes management, weight and weight management, weight loss surgery and participating in weight loss research.

BS was discussed towards the end of the interview, and in order to ensure that participants were informed enough to comment on their willingness to consider BS, interviewees were provided with information on the advantages and disadvantages of surgery (Table 2). To capture participants’ pre-interview stance, interviewees were first asked about their knowledge of and willingness to consider BS before any information was given. This was then re-evaluated once participants had been given the information.

**Table 2:** Bariatric surgery pros and cons

Advantages	Disadvantages
Improved life expectancy	Increased short-term risk of death (low risk 1 in 300 to 1 in 1000 for gastric bypass and band, respectively)
Improved diabetes/possible remission	Risk of post-operative complications
	Hospital stay (1–4 days)
Improved quality of life	Time off work (1–4 weeks)
Less need for medication	Need for lifelong vitamin and mineral supplements
Substantial weight loss	Possible weight regain
Improved fitness	Possible development of loose skin folds
Altered body appearance	Intolerance to certain foods
More control over eating behaviour	May restrict eating out
	Possible diarrhoea or loose/foul smelling stools (gastric bypass)
	Risk of reoperation (one in five over 5 years for gastric band)

The above table was developed on the basis of available literature and author expertise, particularly consultant surgeons J.B. and R.W. Study lay advisors commented on content. R.H.S. sat in weight loss surgery clinic consultations and received training from J.B. to ensure that information provision during interviews was proficient.

Interviews lasted 40–90 min and were digitally recorded and transcribed verbatim. Participants were either interviewed at their GP practice (*n* = 7) or their own home (*n* = 15), depending on their preference, and completed a brief demographic questionnaire that recorded: age, sex, ethnicity, educational level, occupation and social circumstances. From the outset, participants were attributed pseudonyms that were used throughout the study as identifiers, to protect participant anonymity and maintain confidentiality.

### Data analysis

Interviews were analysed in accordance with Framework Analysis as described by Ritchie and Spencer's (1994) ‘Framework’ approach. This method consists of five steps:

1) *Familiarisation*: the analysts read through transcripts and notes, pull out key ideas and start to identify recurrent themes.
2) *Thematic framework*: a framework is constructed on the basis of the original research aims and the recurrent themes identified during the familiarisation process.
3) *Indexing*: the thematic framework is applied consistently to all data.
4) *Charting*: data from the original context are lifted and placed within the appropriate thematic categories within the framework.
5) *Mapping*: the final stage in the process is to interpret and map the range, polarities and similarities within the data.

Negative case analysis, where instances of disagreement are explored, was also used.

### Rigour

During data collection, audio recordings were transcribed within 1–3 days of the original interviews. All team and lay members were sent transcripts and were invited to comment. This was to reduce the chance of important issues raised during interviews being missed or not followed up. It also aided the team in familiarising themselves with the data and in the development of the framework. Lay members were provided with the analysis procedure and training and support was provided where necessary. Before the analysis began, team members were encouraged to consider their own personal perspectives and how this could shape their analysis.

Transcripts were divided among the team (which included lay advisors), with one member, R.H.S., acting as the primary analyst coding all transcripts, and collating comments and codes from other team members. All team members were provided with training and support on the analysis procedure. Regular team meetings and open discussion ensured that the framework development was transparent and credible.

## Results

### Sample characteristics

A total of 22 people were interviewed. Table 3 presents the sample demographics. Of the 22 patients, 19 were white British, two were South Asian and one was white South African. Of the interviewees, 20 controlled their diabetes with oral medication, two were insulin dependent.

**Table 3:** Sample demographic information (*n* = 22)

Demographic	Range (median)
Age (years)	47–73 (64)
Sex (male:female)	13:9
BMI (kg/m^2^)	30–38 (33)
Years diagnosed with type 2 diabetes	3–18 (6)

### Perceptions of BS and willingness to participate in RCT

All participants had some prior awareness of BS, and there was a commonly expressed perception that BS was for ‘those who are more grossly obese’ (Valerie, 73 years), ‘a last resort’ (Harry, 71 years) for people who had ‘a real problem to lose weight’ (Grace, 62 years). It was clear that none of the participants considered themselves to fall within the group at which BS was aimed:

*‘I've never even thought of it [BS]. I don't think I'm that big for me to start going through stuff like this*’

(Ilias, 56 years, anti-BS)

Despite the belief that BS was for larger individuals, there was variable personal interest. Some felt BS was absolutely inappropriate for them, whereas others reported a willingness to consider BS. Table 4 summarises the interest expressed towards BS and weight loss research. All interviewees who were pro-BS or unsure-BS were open to considering participating in an RCT. Conversely, those who were anti-BS were largely unwilling to participate in RCT research, in case they ‘ended up having surgery’ (Grace, 62 years, anti-BS), and those who did say they would take part explained that if they were put in the surgery group they would ‘be walking out’ of the trial (Bernard, 47 years, anti-BS). Noteworthy, the four participants underscored in Table 4 had initially rejected the idea of BS before being presented with the BS risk and benefit information and altered their stance afterwards. Participants reported having been told that type 2 diabetes was a lifelong condition, believing that ‘once you've got diabetes, you've got diabetes’ (Ilias, 56 years, anti-BS), and these interviewees expressed surprised, and in some cases scepticism, as to whether BS could result in remission of type 2 diabetes.

**Table 4:** Interest towards BS and research [Patient (BMI kg/m^2^ and treatment)]

Participant stances	Would consider BS	Would not consider BS	Unsure about BS
Would consider RCT	Arnold (32/M), Charlotte (37/M), Joyce (34/M), Ruth (36/M)	Bernard (34/M), Grace (31/M)	Felicity (31/M), Marcus (31/M), Nathan (38/M), Sophie (37/M), Phil (38/M)
Would consider preference trial only		Dennis (31/M), Leonard (30/I), Harry (30/M), Ian (32/M), Oliver (38/M), Trevor (32/I), Valerie (32/M), Sashi (35/M), Ilias (35/M)	
No interest in interventional weight loss research		Kath (30/M), Edward (31/M)	

BS = bariatric surgery; M = oral medication, I = insulin therapy

When discussing the reasons for an interviewee's particular stance, a number of considerations arose. Disadvantages to surgery such as increased risk of death, infection, post-operative complications and the permanent nature of surgery were all highlighted as off-putting, both by those not interested in BS and those unsure of BS. Conversely, some of those interested in BS reported being less concerned by the above, noting that such risks were to be expected with any kind of surgery. Two themes emerged as influential when determining the individual's stance on the appropriateness of BS: ‘condition-related life impact’ and ‘perceived control’.

### Condition-related life impact

All patients interviewed recognised the importance of both diabetes and obesity. However, despite this recognition, there was great variance in the extent to which individuals felt their diabetes or weight was a threat to their own health and well-being.

Some interviewees reported having adopted a healthier lifestyle despite being asymptomatic, with the desire to avoid related complications or injections being highlighted as motivational. Conversely, for others, the absence of complications appeared to reduce the perception of threat and subsequent desire to adopt healthier lifestyles. Ian (72 years) explained that as he had not experienced any problems, this, coupled with his philosophy of ‘never try jumping fences that aren't there’, meant that having diabetes did not motivate him to alter his lifestyle and others commented comparably. Lack of symptoms was perceived by some as evidence that their diabetes was under control and well managed, irrespective of their weight status or HBA1c.

In contrast, other participants had experienced complications, which they contextualised in terms of the impact these complications had on their day-to-day living. For example, one female respondent explained that the fatigue she experienced as a consequence of diabetes diminished her ability to both plan and attend social events with friends, and do pastimes such as gardening:

*‘I'm putting it down to the diabetes, but the fact that I wake up extremely tired and exhausted, and I can't get through the day, even though I've had a good night's sleep*’

(Felicity, 70 years, unsure-BS)

For some, excess weight was described as more influential. This was particularly discussed among those who attributed their own physical limitations to weight-related health problems, though aesthetic dissatisfaction was also reported:

‘*My weight? Oh, I want to lose it. Oh, I'm desperate because that would ease the weight on my knees and therefore I'd be able to walk further*’

(Joyce, 67 years, pro-BS)

‘*Well, I'm not too happy with it. You know, I sometimes look in a mirror and I call myself all kinds of names, fat so-and-so and everything else*’

(Nathan, 64 years, unsure-BS)

The impact of diabetes and obesity was variable, with only some individuals experiencing problems. Individuals with no interest in BS, such as Kath (72 years, anti-BS), Ilias (56 years, anti-BS) and Oliver (67 years, anti-BS), appeared to share the characteristic of being relatively asymptomatic. These individuals felt that neither their diabetes nor their weight adversely affected their lives. Given the absence of disease-related symptoms or functional impairments, this group of interviewees questioned how BS would be of any benefit. This inability to perceive potential benefit was contrasted with the invasive nature of BS and potential harm associated with surgery and hospitalisation. As a result, some concluded ‘if you don't need to then don't bother’ (Ilias, 56 years, anti-BS). Conversely, where participants reported one or both conditions to be negatively affecting their lives, BS was seen as having the potential to convey benefits and as such, worth considering:

‘*It's [BS] got to improve your fitness, if you're not carrying all that weight around. I put a lot of that down to why I can't walk very far without getting out of breath*’

(Charlotte, 50 years, pro-BS)

### Perceived control

There were individuals such as Valerie (73 years), Trevor (55 years) and Grace (62 years) who did feel that their diabetes and weight influenced their lives and yet felt that BS was inappropriate in their management. A key difference between these individuals and other interviewees such as Charlotte (50 years), Joyce (67 years), Felicity (70 years) and Nathan (64 years), who were interested in BS, was their perception of control. Those feeling able to alter their situation did not feel that BS was necessary, whereas those who felt unable to exert change were more willing to consider BS. Table 5 compares Trevor and Felicity's contrasting situations.

**Table 5:** Comparative quotes

(1a) Trevor (55 years, anti-BS) on his ability to control his weight	(1b) Felicity (73 years, unsure-BS) on her ability to lose weight
*Well I was 107 kilos and I am now as of this morning just over 90, so that's 17 kilos in just a year.*	*‘I can't seem to lose any weight. I have tried several diets. I've even tried not eating, and I just don't lose an ounce’.*
(2a) Trevor (55 years, anti-BS) on his interest in BS	(2b) Felicity (73 years, unsure-BS) on her attitude towards BS
*I wouldn't have either of them done (band or bypass). I hope I stay in control. Who knows in 10 years from now, you don't know do you, your circumstances, but at the moment in my life I feel very much in control of my body which is a help I think.*	‘Because I would really like to lose weight, and if I could be assured – it's difficult really to say. I would consider –I possibly would consider surgery’

It should be noted that not all participants who lacked the ability to control their weight were pro-BS. Indeed, Sophie (63 years, unsure-BS) described how she would find it difficult to consider BS, as she felt that the diabetes was her ‘fault’ and that she ‘should be able to control it, I'm a grown woman after all! I'm not an idiot of a child’. Sophie (63 years, unsure-BS) concluded by saying that her feelings surrounding her weight and self-responsibility were ‘almost like a moral thing’ and that she found it difficult to consider BS because she felt that she ‘shouldn't need that [BS]’.

For some, perception of control was informed by the individual's expectations of ageing, with both health problems and increased weight being associated with growing older. Moreover, whereas some such as Felicity (73 years, unsure-BS) were still willing to consider BS, others who were not unhappy with their situation questioned whether any benefits derived from BS would be enjoyed for a sufficient length of time so as to warrant the short-term inconveniences and increase risk of mortality associated with undergoing surgery:

‘*At my time in life 72, nearly 73 I feel the improvement isn't going to be as long term as for someone in their 30's, 40's, 50's. So I think the scales come down more on the status quo rather than wanting to change it by taking that road*’

(Ian, 72 years, anti-BS)

Noteworthy, among those who were not in favour of BS, some were willing to consider it as an option in the future if their circumstances were to change, such as their health worsening considerably or losing control:

‘*If somebody turned round to me and said ‘if you don't have this operation done [BS], you'll be dead in three months’ time’, you would seriously contemplate it, wouldn't you, because you then look at the priority and turn round and say ‘okay, on balance, I'll have it done’ so I think that's about the only time. It's when it comes down to something being critical. I don't think that my situation is critical*’

(Oliver, 67 years, anti-BS)

‘*If I didn't have the willpower and I was offered it [BS], yes. I think it's a great thing to help people. Well I hope I stay in control. Who knows in 10 years from now, you don't know do you, your circumstances, but at the moment in my life I feel very much in control of my body which is a help I think*’

(Trevor, 55 years, anti-BS)

## Discussion

Previous studies have commonly explored the perspectives of more severely obese individuals who have already undergone BS (Ogden *et al*., 2006; Munoz *et al*., 2007; Karmali *et al*., 2011). This study is unique, presenting the attitudes of an under-researched population towards BS; those who are non-morbidly obese, with type 2 diabetes; and those who have not had BS. Key findings were that some patients considered BS to be an acceptable intervention, and would consider participating in an RCT. Those interested in BS had a range of BMIs, suggesting that other factors also influenced their interest in surgery. Our findings suggest that both the extent to which people's lives are negatively affected by their weight and/or diabetes, and their perceived control may be significant.

Those interested in BS and even those unsure of BS identified beneficial factors associated with BS, including improved diabetes control, increased mobility, reduced joint pain, altered physical appearance and substantial weight loss. In contrast, those who were not interested in BS reported being less affected by their weight or diabetes and as such did not feel BS conveyed any particular benefits. This suggests that where diabetes or weight are perceived to have an impact on life intolerably, such as imposing restrictions to participation in valued activities, individuals may be more likely to consider BS. This supports previous research into motivations for BS in populations with higher BMIs (Ogden *et al*., 2006; Munoz *et al*., 2007; Karmali *et al*., 2011). Given the current economic climate and funding limitations for health-care interventions, it is most likely that future studies into the effects of BS in this population will investigate the economic benefit of BS for patients who have the highest absolute risk of adverse outcomes and health-care costs, including those starting on insulin or with diabetic complications. As such, it is plausible that this subgroup of non-morbidly obese patients with T2DM may identify negative condition-related impact higher and are more likely to identify a positive role for BS in their management.

Importantly, some individuals did not perceive their weight or diabetes to affect their health, considering their lack of symptom testament to this. That individuals may not be compelled to alter their lifestyle or lose weight until symptomatic has been reported elsewhere (Murphy and Kinmonth, 1995; Brown *et al*., 2002; Matthews *et al*., 2009; Zhang *et al*., 2011). A related issue concerns the perception that type 2 diabetes is a permanent, irreversible condition. Some authors note that this perception may deter patients from implementing positive lifestyle changes (Murphy and Kinmonth, 1995). Furthermore, it may also prevent such patients perceiving BS as a potentially appropriate intervention for type 2 diabetes. Clinicians need to be aware that information regarding BS may contradict their patients’ established beliefs surrounding the permanency of diabetes and may need to be targeted educationally.

Negative condition-related impact on life was not always indicative of interest in surgery. Some patients did feel their diabetes and/or weight to be detrimental but were still unwilling to consider BS. In such cases, the individual's perception of control was identified as moderating interest. Those who felt they were able to exert control over their weight and/or diabetes seemed less open to considering BS, as were those who described being unbothered by their condition. In contrast, those who felt they were unable to control their weight and were unhappy about this seemed likelier to consider BS. This finding complements work by Ogden *et al*. (2006), who found that patients undergoing BS reported feeling a renewed sense of control. Our findings suggest an exception to this, where the individual feels a strong sense of responsibility towards their own weight and is unable to relinquish control because of this. This supports Brown *et al*.'s (2006) suggestion that high levels of self-responsibility and consequent embarrassment may deter patients who are obese from accessing primary care support for weight management issues, and underscores its potential to deter patients from considering BS specifically. In such cases, health professional recommendation may alleviate the sense of guilt arising from personal perceptions of having failed to exert a ‘proper’ degree of self-control. Age seemed influential to some patients’ attitudes, with some potentially feeling too old to derive enough benefit to personally justify the time and effort involved in undergoing BS.

All participants who were in favour or unsure of BS were willing to consider participating in RCTs comparing BS with a non-surgical alternative. Some participants had altered their stance after considering the risk and benefit information, which suggests that the provision of information can alter perceptions in favour of BS for some individuals. Conversely, those unwilling to consider BS were unwilling to participate in RCTs for fear of being allocated BS. However, most participants were willing to take part in a preference trial, which reflected a general interest in weight reduction.

This study followed a robust methodological design and utilised peer review, multiple coders and negative case analysis to produce a credible piece of research. Although the results presented were derived from a sample whose demographics adequately represented the sexes and were diverse in ages, BMI and years diagnosed, having a predominantly white British sample, most of whom were orally medicated, was a limitation. Future research considering other ethnic groups and treatment regimens may identify further influential factors, thereby building on this work. Moreover, the findings presented are *suggestive* of factors that influence a patient's willingness to consider BS and participate in an RCT. The overall support for BS needs to be determined in an adequately sized survey, in a representative group of patients and identified hypotheses tested. The findings here will guide the development of a questionnaire survey into patient attitudes towards BS and willingness to participate in a research trial comparing BS with a non-surgical intervention.

## Conclusion

This study found diverse attitudes towards BS among non-morbidly obese patients with type 2 diabetes. BS may be an acceptable option to patients who both experience negative effects as a consequence of their diabetes and/or excess weight, and struggle with weight/glycaemic control, although age expectations may also be influential. In contrast, people who do not perceive their condition (weight or diabetes) to negatively affect their lives and feel able to control their weight/diabetes may be less inclined to consider BS as a viable option. The provision of adequate, balanced information on BS may encourage some individuals to re-evaluate their stance towards BS. Although most would consider participating in weight management research, those interested in BS were more willing to consider participating in an RCT.
